# Novel mutations in penicillin-binding protein genes in clinical *Staphylococcus aureus* isolates that are methicillin resistant on susceptibility testing, but lack the *mec* gene

**DOI:** 10.1093/jac/dkt418

**Published:** 2013-11-11

**Authors:** Xiaoliang Ba, Ewan M. Harrison, Giles F. Edwards, Matthew T. G. Holden, Anders Rhod Larsen, Andreas Petersen, Robert L. Skov, Sharon J. Peacock, Julian Parkhill, Gavin K. Paterson, Mark A. Holmes

**Affiliations:** 1 Department of Veterinary Medicine, University of Cambridge, Cambridge, UK; 2 Scottish MRSA Reference Laboratory, NHS Greater Glasgow and Clyde, Stobhill Hospital, Glasgow, UK; 3 Wellcome Trust Sanger Institute, Hinxton, UK; 4 Department of Microbiology and Infection Control, Statens Serum Institut, Copenhagen, Denmark; 5 Department of Clinical Medicine, University of Cambridge, Cambridge, UK

**Keywords:** β-lactams, MRSA, *mecA*, *mecC*

## Abstract

**Objectives:**

Methicillin-resistant *Staphylococcus aureus* (MRSA) is an important global health problem. MRSA resistance to β-lactam antibiotics is mediated by the *mecA* or *mecC* genes, which encode an alternative penicillin-binding protein (PBP) 2a that has a low affinity to β-lactam antibiotics. Detection of *mec* genes or PBP2a is regarded as the gold standard for the diagnosis of MRSA. We identified four MRSA isolates that lacked *mecA or mecC* genes, but were still phenotypically resistant to pencillinase-resistant β-lactam antibiotics.

**Methods:**

The four human *S. aureus* isolates were investigated by whole genome sequencing and a range of phenotypic assays.

**Results:**

We identified a number of amino acid substitutions present in the endogenous PBPs 1, 2 and 3 that were found in the resistant isolates but were absent in closely related susceptible isolates and which may be the basis of resistance. Of particular interest are three identical amino acid substitutions in PBPs 1, 2 and 3, occurring independently in isolates from at least two separate multilocus sequence types. Two different non-conservative substitutions were also present in the same amino acid of PBP1 in two isolates from two different sequence types.

**Conclusions:**

This work suggests that phenotypically resistant MRSA could be misdiagnosed using molecular methods alone and provides evidence of alternative mechanisms for β-lactam resistance in MRSA that may need to be considered by diagnostic laboratories.

## Introduction

β-Lactam antibiotics work by acylation of the transpeptidase domain active site of penicillin-binding proteins (PBPs), blocking access to their substrate and preventing cross-linking of peptidoglycan strands during cell wall synthesis. Initially, resistance to penicillin in *Staphylococcus aureus* was mediated by the expression of a β-lactamase enzyme, which breaks down penicillin. Since the introduction of methicillin, a semi-synthetic β-lactamase-resistant penicillin, in 1961, methicillin-resistant *S. aureus* (MRSA) has emerged that is resistant to the majority of β-lactam antibiotics.^1^ Resistance to β-lactams in MRSA is mediated by the acquisition of the *mecA* gene, which encodes an alternative PBP2a, which has low affinity for β-lactam antibiotics and enables the bacteria to assemble the cell wall in the presence of the drug.^2,3^ More recently, a divergent form of the *mecA* gene, known as *mecC* (previously *mecA*_LGA251_), was identified in isolates of *S. aureus* from both animals and humans.^4^ *S. aureus* isolates with other types of resistance to β-lactams have been described and are known as borderline oxacillin-resistant *S. aureus* (BORSA) or intrinsically resistant *S. aureus*.^5,6^ Resistance in some BORSA isolates is attributed to the presence or overexpression of β-lactamase enzymes,^6,7^ while in intrinsically resistant and some BORSA isolates it is mediated by chromosomal mutations.^5,8,9^ Furthermore, *in vitro* studies generating β-lactam-resistant isolates under the selection of β-lactam antibiotics have also identified a number of other genes able to mediate intrinsic β-lactam resistance.^8,10,11^ Here, we report four clinical MRSA isolates from three multilocus sequence types (STs) that are resistant to β-lactamase-resistant penicillins, but lack both *mecA* and *mecC*. This demonstrates that *mec*-negative MRSA isolates with resistance to multiple β-lactams may be responsible for human infection, but may be misclassified as methicillin susceptible based on detection of *mecA/C* or PBP2a, potentially leading to treatment failure.

## Methods

Isolates were grown on blood agar (Oxoid, UK) and in tryptone soya broth (TSB) at 37°C. A list of the isolates used in this study is shown in Table 1. Antimicrobial susceptibility testing was performed using disc susceptibility testing according to BSAC criteria (BSAC Methods for Antimicrobial Susceptibility Testing, version 11.1, May 2012). Strains NCTC 6571, NCTC 12493, LGA251 and NCTC 29213 acted as susceptible, *mecA* resistant, *mecC* resistant and β-lactamase overproducer controls, respectively. Isolates were tested for β-lactamase production by using β-lactamase (nitrocefin) identification sticks (Oxoid, UK) according to the manufacturer's instructions. The cloverleaf assay was carried out as previously described.^12^ Genomic DNA was extracted from overnight cultures grown in TSB at 37°C using a MasterPure Gram-positive DNA Purification Kit (Cambio, UK). Illumina library preparation and Hi-Seq sequencing were carried out as previously described.^13^ Orthologous proteins were identified using BLAST.

**Table 1.** Relevant phenotypic and genotypic characteristics of resistant isolates

Isolate	Country	Origin	Isolated from	Year of isolation	MLST	*spa* type	OXA (mm)	FOX (mm)	OXA Etest (mg/L)	FOX Etest (mg/L)	PEN G (mm)	PEN G Etest (mg/L)	CRO Etest (mg/L)	β-Lactamase	*blaZ* gene	*blaZ* type
XB84	Scotland	human	wound	2010	15	t084	11	17	2	12	9	2	>32	+	+	C
XB85	Scotland	human	wound	2010	1	t127	0	18	4	6	12	1	>32	+	+	C
XB86	Scotland	human	wound	2010	15	t907	0	17	3	8	19	0.25	>32	−	−	−
XB87	Scotland	human	wound	2010	8	t008	0	12	4	16	10	1.5	>32	+	+	A

	PEN G, 1 μg	PEN G + CLA, 0.5 μg	PEN G + CLA, 1 μg	OXA, 1 μg	OXA + CLA, 0.5 μg	OXA + CLA, 1 μg	FOX, 10 μg	FOX + CLA, 5 μg	FOX + CLA, 10 μg	CLA, 0.5 μg	CLA, 1 μg	CLA, 2 μg	CLA, 5 μg	CLA, 10 μg		
XB84	9	11	12	11	12	12	17	18	18	0	0	0	0	0		
XB85	12	13	14	0	0	0	18	20	20	0	0	0	0	0		
XB86	18	19	20	0	0	0	17	18	19	0	0	0	0	7		
XB87	10	15	15	0	0	0	12	15	15	0	0	0	0	7		

MLST, multilocus ST; OXA, oxacillin; FOX, cefoxitin; PEN G, penicillin G; CRO, ceftriaxone; CLA, clavulanic acid.

For oxacillin: Etest MIC breakpoint is >2 mg/L; disc diffusion—susceptible ≥15 mm diameter, resistant ≤14 mm diameter.

For cefoxitin: Etest MIC breakpoint is >4 mg/L; disc diffusion—susceptible ≥22 mm diameter, resistant ≤21 mm diameter.

For penicillin G: Etest MIC breakpoint is >0.12 mg/L; disc diffusion—susceptible ≥25 mm diameter, resistant ≤24 mm diameter.

## Results

We identified four *mecA*/*C*-negative isolates (XB84, 85, 86 and 87) from the Scottish MRSA Reference Laboratory that exhibited resistance to penicillinase-resistant β-lactams*.* All four were isolated from human wound infections in Scotland and belonged to STs 15, 1, 15 and 8, respectively. All four isolates exhibited resistance to oxacillin, cefoxitin and penicillin by both Etest and disc diffusion assays (BSAC, version 11.1) (Table 1) (except XB84, which was at the oxacillin Etest breakpoint). All four isolates also had an MIC of ceftriaxone of >32 mg/L (Table 1). Three isolates (XB84, 85 and 87) were positive for β-lactamase production as measured by β-lactamase (nitrocefin) sticks and a cloverleaf assay. To further elucidate the molecular basis of resistance, the four isolates underwent whole genome sequencing. This confirmed that none of the isolates harboured a *mecA* or *mecC* gene, or any coding sequence resembling a putative *mec* gene homologue. Bioinformatic analysis confirmed that the isolates (XB84, 85 and 87) that were positive for β-lactamase production had a *blaZ* gene and that XB86, which was negative for β-lactamase production, lacked *blaZ* (Table 1). Further analysis of the *blaZ* genes in each isolate showed that XB87 had a type A *blaZ* gene, while both XB84 and XB85 had an identical (100% nucleotide identity) type C *blaZ* gene.^14^

We next used BLAST to identify further isolates in our sequenced genome collection that shared 100% nucleotide identity across the entire β-lactamase (*bla*) locus (*blaZ*-*blaI*-*blaR*) for the two *blaZ* genes (types A and C). We identified three isolates with a type A *blaZ* gene and 12 isolates with a type C *blaZ* gene and tested them for phenotypic resistance to oxacillin and cefoxitin (data not shown). No association was found between the presence of either type of *blaZ* gene and resistance. To further investigate the role of *blaZ* in the resistant isolates, we repeated the disc diffusion assays in the presence of clavulanic acid (a β-lactamase inhibitor) at ratios of 2 : 1 and 1 : 1, antibiotic : clavulanic acid. No major reduction was seen in the zones of inhibition of oxacillin, cefoxitin or penicillin in combination with clavulanic acid at either 2 : 1 or 1 : 1, suggesting that β-lactamase production was not mediating resistance (Table 1).

In addition to PBP2a and β-lactamase, five other proteins (PBP2, PBP4, GdpP, YjbH and AcrB) have been reported previously to be associated with β-lactam resistance in *S. aureus.*^8–11,15–17^ We screened the genome sequences of the four resistant isolates for the presence of previously reported mutations, but none was found in our isolates. Next, we compared the sequences for these same five proteins from the four resistant isolates with a panel of susceptible isolates belonging to the same STs (ST1, 10 isolates; ST8, 4 isolates; and ST15, 2 isolates) (data not shown). In the comparison, we also included the two other endogenous PBPs (PBP1 and PBP3) on the basis that these are also bound by β-lactam antibiotics.^17,18^ We found a small number of amino acid substitutions to be present in PBP1, PBP2, PBP3 and YjbH in one or more of the resistant isolates, but absent in the susceptible counterparts (Table 2). Interestingly, we found three separate residues with substitutions that were present in at least two isolates from different STs, suggestive of homoplasy (independent evolution of the same trait). First, a His-499→Tyr substitution in the transpeptidase domain of PBP1 was present in three isolates from three different STs: XB85 (ST1), XB86 (ST15) and XB87 (ST8) (Table 2). Second, a Thr-552→IIe substitution in the transpeptidase domain of PBP2 was found in three isolates from two STs: XB84 (ST15), XB86 (ST15) and XB87 (ST8). Third, isolates XB84 (ST15), XB85 (ST1) and XB86 (ST15) all shared the same Ser-634→Phe substitution in the transpeptidase domain of PBP3 (Table 2). Fourth, we identified two different non-conservative amino acid substitutions at the same position in the transpeptidase domain of PBP1, a Tyr-336→Asn substitution in XB84 (ST15) and a Tyr-336→Cys substitution in XB85 (ST1). A number of single substitutions were also present in one of the resistant isolates that were absent in the susceptible isolates of the same ST (Table 2). Two further substitutions were present in PBP2: a Thr-31→Met substitution in XB85 and an Asp-156→Tyr substitution in the transpeptidase domain of isolate XB86. XB85 also had a Thr-371→IIe substitution in the transpeptidase domain of PBP1 (Table 2). Except for MRSA strain CM05 (accession: AMAB00000000), which had the Asp-156→Tyr mutation in PBP2, we were unable to find any genomes in the NCBI/EMBL databases containing the same substitutions.

**Table 2.** Locations of amino acid substitutions identified in methicillin-resistant isolates that were absent in susceptible isolates of the same ST

		Protein									
		PBP1				PBP2			PBP3		YjbH
Isolate	MLST	Y336C	Y336N	T371I	H499Y	T31M	D156Y	T552I	Y430D	S634F	D137N
XB84	15	−	+	−	−	−	−	+	−	+	−
XB85	1	+	−	+	+	+	−	−	−	+	−
XB86	15	−	−	−	+	−	+	+	−	+	+
XB87	8	−	−	−	+	−	−	+	+	−	−

MLST, multilocus ST.

## Discussion

In this work we have identified MRSA isolates belonging to three STs, which unlike BORSA strains are resistant to both oxacillin and cefoxitin. This resistance does not appear to be mediated by hyperproduction of a β-lactamase and we have identified a number of novel substitutions in the transpeptidase domains of PBPs 1, 2 and 3 that we hypothesize could mediate this resistance. The transpeptidase domains of PBPs are the target of β-lactam antibiotics and substitutions in the transpeptidase domain of PBP2 have been shown previously to reduce the acylation efficacy of PBP2 by β-lactams and thus provide some degree of resistance.^8,15^ Given the small number of isolates in this study and that our targeted search for mutations might have excluded other genes involved in resistance, further experimental work is necessary to characterize the contribution of the novel PBP substitutions to β-lactam resistance. Mutations in PBP1 and PBP3 have not been implicated previously in *S. aureus* resistance; however, in *Staphylococcus lugdunensis*, a tetrapeptide duplication in the transpeptidase domain of PBP1A/B has been shown to be associated with increased β-lactam resistance.^19^ Currently, *mec* gene-negative MRSA isolates are not widely reported, but it is important to characterize the basis for resistance in these isolates, especially for clinical laboratories that rely on the molecular detection of *mecA*/*C* and/or PBP2a as the gold standard for MRSA detection. Furthermore, PBP2a has been highlighted as an attractive target for drug development^20^ and should PBP2a-targeted inhibitors become available, it is important to understand alternative mechanisms for *S. aureus* to develop resistance to β-lactams. Finally, it is clear that there are multiple distinct mechanisms for β-lactam resistance in *S. aureus* and these need to be taken into consideration by diagnostic laboratories.

## Funding

This work was supported by a Medical Research Council Partnership grant (G1001787/1) held between the Department of Veterinary Medicine, University of Cambridge (M. A. H.), the School of Clinical Medicine, University of Cambridge (S. J. P.), the Moredun Research Institute and the Wellcome Trust Sanger Institute (J. P. and S. J. P.). S. J. P. receives support from the NIHR Cambridge Biomedical Research Centre. X. B. was supported by the China Scholarship Council and the Cambridge Overseas Trust.

## Transparency declarations

None to declare.
